# Immunotherapy in Endometrial Cancer: Molecular Classification, Clinical Evidence, and Therapeutic Implications: A Narrative Review

**DOI:** 10.3390/cancers18111709

**Published:** 2026-05-24

**Authors:** Pablo Padilla-Iserte, Silvia Cabrera, Sonia Gatius Calderó, Ana de Juan Ferré, Katarina Majercakova, María Jesús Rubio-Pérez, Ignacio Romero, Maria Pilar Barretina-Ginesta, Manel Barahona Orpinell

**Affiliations:** 1 Department of Gynecologic Oncology, La Fe University and Polytechnic Hospital, Instituto de Investigación Sanitaria La Fe (IISLAFE), 46026 Valencia, Spain; 2 Department of Pediatrics, Obstetrics and Gynaecology, University of Valencia, 46010 Valencia, Spain; 3 Department of Gynecologic Oncology, Hospital de la Santa Creu i Sant Pau, Institut de Recerca Sant Pau (IR SANT PAU), Universitat Autònoma de Barcelona, 08041 Barcelona, Spain; 4 Department of Pathology, Arnau de Vilanova University Hospital, Institut de Recerca Biomèdica de Lleida, University of Lleida, 25198 Lleida, Spain; 5 Department of Medical Oncology, Marqués de Valdecilla University Hospital, Instituto de Investigación Marqués de Valdecilla (IDIVAL), 39008 Cantabria, Spain; 6 Department of Radiation Oncology, Hospital de la Santa Creu i Sant Pau, 08041 Barcelona, Spain; 7 Department of Medical Oncology, Reina Sofía University Hospitalv Córdoba, 14004 Córdoba, Spain; 8 Department of Medical Oncology, Valencian Institute of Oncology (IVO) Foundation, 46009 Valencia, Spain; 9 Medical Oncology Department, Institut Català d’Oncologia, Girona Biomedical Research Institute (IDIBGI-CERCA), Girona University, 17007 Girona, Spain; 10 Department of Gynecologic Oncology, University Hospital Limerick, Mid-West HSEl, V94 F858 Limerick, Ireland

**Keywords:** endometrial cancer, immunotherapy, immune checkpoint inhibitors, PD-1/PD-L1, molecular classification, mismatch repair deficiency

## Abstract

Background/Objectives: Endometrial cancer (EC) is the most common gynecologic malignancy in developed countries, with increasing incidence and limited options in advanced disease. Molecular classification has redefined risk stratification and therapeutic decision-making, particularly with the incorporation of immunotherapy. This review provides a clinically oriented overview of immunotherapy in EC across molecular subgroups and treatment settings. Methods: A narrative review was conducted using PubMed/MEDLINE, Embase, and Web of Science, focusing on clinical trials and studies with direct clinical relevance. Results: Immune checkpoint inhibitors targeting the PD-1/PD-L1 axis have demonstrated significant benefit in EC, particularly in mismatch repair-deficient (dMMR)/microsatellite instability–high (MSI-H) tumors, where durable responses are observed. In contrast, mismatch repair-proficient (pMMR) tumors show limited sensitivity to monotherapy and require combination approaches. Recent phase III trials have established chemoimmunotherapy as a first-line standard, with greater benefit in dMMR tumors and clinically meaningful improvements in pMMR disease. In the second-line setting, PD-1 inhibitor monotherapy is standard for dMMR tumors, while lenvatinib plus pembrolizumab is a key option for pMMR disease. However, responses remain heterogeneous and are not fully explained by MMR status alone. Conclusions: Immunotherapy is a cornerstone in advanced EC management, guided by molecular classification. Key challenges include limited efficacy in pMMR tumors, lack of robust predictive biomarkers, and uncertainty in treatment sequencing. Future strategies should focus on biomarker-driven approaches and rational combinations.

## Simple Summary

Endometrial cancer is the most common gynecologic malignancy in developed countries, with a rising incidence. While early-stage disease is often curable, advanced and recurrent cases remain challenging to treat. Immunotherapy, particularly PD-1/PD-L1 checkpoint inhibitors, has improved outcomes in selected patients. Molecular classification, especially mismatch repair (MMR) status, is essential to guide treatment: MMR-deficient tumors respond well to immunotherapy, whereas MMR-proficient tumors show limited benefit and often require rational combination strategies. Recent clinical trials have established chemoimmunotherapy as a new standard for advanced disease. However, responses remain heterogeneous, and factors such as the tumor microenvironment and additional biomarkers are increasingly recognized as key determinants of treatment efficacy. Ongoing research aims to refine patient selection and develop more effective therapeutic strategies.

## 1. Introduction

Endometrial cancer (EC) is the most common gynecologic malignancy in developed countries, and its global incidence continues to rise, largely driven by increasing obesity rates, population aging, and associated metabolic disorders [1,2]. Although most patients are diagnosed with early-stage disease and can be effectively treated with surgery, advanced and recurrent EC continue to represent a major therapeutic challenge due to limited treatment options and unfavorable clinical outcomes. For many years, platinum-based chemotherapy, particularly the combination of carboplatin and paclitaxel, has remained the cornerstone of first-line treatment in this setting despite providing only modest survival benefit and limited durability of response.

During the last decade, the therapeutic landscape of EC has evolved substantially through two major advances: the incorporation of molecular classification into routine clinical practice and the development of immunotherapy-based strategies. The molecular classification proposed by The Cancer Genome Atlas (TCGA) in 2013 established EC as a biologically heterogeneous disease composed of four distinct molecular subgroups: *POLE* ultramutated, microsatellite instability–hypermutated, copy-number low, and copy-number high (serous-like) tumors [3]. This framework has progressively transformed risk stratification and therapeutic decision-making and is currently recommended as part of standard diagnostic assessment [4] (Table 1).

Among these molecular subgroups, tumors harboring mismatch repair deficiency (dMMR) or microsatellite instability–high (MSI-H) status are characterized by a high tumor mutational burden and increased neoantigen generation, resulting in an inflamed tumor microenvironment and enhanced sensitivity to immune checkpoint blockade [2]. These tumors typically exhibit increased tumor-infiltrating lymphocytes, interferon-γ signaling, and upregulation of immune checkpoints such as PD-1 and PD-L1, which partly explains their favorable responsiveness to immunotherapy. In contrast, mismatch repair-proficient (pMMR) tumors are generally less immunogenic, display lower levels of immune infiltration, and show more heterogeneous biological and clinical behavior. Consequently, pMMR disease remains a major therapeutic challenge, with lower response rates to single-agent immunotherapy and a greater need for rational combination strategies aimed at overcoming primary immune resistance.

**Figure 1:**
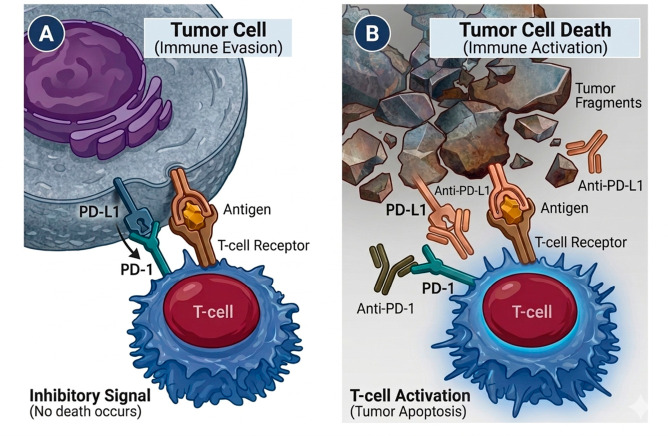
PD-1/PD-L1 immune checkpoint signaling and therapeutic blockade. (**A**) In the absence of immune checkpoint inhibition, PD-L1 expressed on tumor cells binds to PD-1 receptors on activated T cells, generating an inhibitory signal that suppresses T-cell activation and promotes tumor immune evasion despite antigen recognition. (**B**) Blockade of the PD-1/PD-L1 interaction with anti-PD-1 (green) or anti-PD-L1 (yellow) monoclonal antibodies restores T-cell activation and antitumor immune responses, leading to tumor cell apoptosis and release of tumor-associated antigens. Although this simplified schematic focuses on the tumor cell–T-cell interaction, additional components of the tumor microenvironment (including regulatory T cells, myeloid-derived suppressor cells, and cytokine signaling) may also influence response and resistance to immune checkpoint inhibitors.

The introduction of immune checkpoint inhibitors (ICIs), particularly agents targeting the PD-1/PD-L1 axis, has substantially expanded the therapeutic landscape of EC [5,6,7] (Figure 1, Table 2). These therapies have demonstrated durable clinical benefit in biomarker-driven populations and, more recently, have been incorporated into first-line treatment in combination with platinum-based chemotherapy following the results of pivotal phase III trials [8,9,10]. Nevertheless, the clinical benefit remains heterogeneous and cannot be fully explained by MMR status alone. Increasing evidence suggests that additional factors (including tumor microenvironment composition, immune cell infiltration, cytokine signalling, genomic alterations, and alternative immune regulatory pathway) may significantly influence treatment response and resistance.

Furthermore, immunosuppressive components of the tumor microenvironment, including regulatory T cells (Tregs), myeloid-derived suppressor cells (MDSCs), and tumor-associated macrophages, may contribute to immune evasion and reduced sensitivity to ICIs, particularly in pMMR tumors.

These limitations have fueled the development of novel therapeutic strategies combining immunotherapy with chemotherapy, antiangiogenic agents, PARP inhibitors, and other targeted approaches in an attempt to enhance antitumor immunity and broaden clinical benefit across molecular subgroups. Therefore, a deeper understanding of tumor molecular characteristics, immune microenvironment dynamics, and resistance mechanisms will be essential to optimize patient selection, refine treatment sequencing, and guide the future evolution of precision immunotherapy in EC.

## 2. Methods

### 2.1. Study Design

This narrative review was conducted to summarize and critically evaluate the current evidence regarding the role of immunotherapy in EC, with particular emphasis on molecular classification, therapeutic strategies, and their implications for clinical practice.

### 2.2. Literature Search Strategy

A comprehensive literature search was performed using PubMed/MEDLINE, Embase, and Web of Science to identify relevant studies published up to January 2026. The search strategy combined Medical Subject Headings (MeSH) and free-text terms related to endometrial cancer and immunotherapy, including “endometrial cancer”, “endometrial carcinoma”, “immunotherapy”, “immune checkpoint inhibitors”, “PD-1”, “PD-L1”, “dMMR”, “MSI-H”, “molecular classification”, “pMMR”, “PARP inhibitors”, and “targeted therapy”.

To ensure inclusion of the most recent clinically relevant evidence, abstracts and presentations from major international oncology congresses, including ASCO, ESMO, and ESGO meetings, were also reviewed. In addition, the reference lists of selected publications were manually screened to identify additional pertinent studies.

### 2.3. Study Selection

Studies were selected according to their clinical relevance and contribution to the objectives of this review. Eligible publications included phase I–III clinical trials, meta-analyses, systematic reviews, and key observational studies evaluating immunotherapy strategies in endometrial cancer. Particular emphasis was placed on pivotal clinical trials that have influenced contemporary treatment paradigms, especially studies investigating immune checkpoint inhibitors across different disease settings and molecular subgroups.

Preclinical studies were also considered when relevant for understanding biological mechanisms, resistance pathways, or the rationale supporting rational combination strategies. Publications not available in English and studies considered to have insufficient methodological quality or limited clinical relevance were excluded.

### 2.4. Data Extraction and Synthesis

Data extraction was performed by the authors and focused on study characteristics, patient populations, molecular classification, therapeutic strategies, efficacy outcomes, and safety profiles, including objective response rate, progression-free survival, overall survival, and treatment-related adverse events.

Given the narrative nature of this review, no formal meta-analysis or quantitative synthesis was conducted. The available evidence was qualitatively analyzed and organized according to clinical setting (first-line treatment, recurrent disease, and emerging therapeutic strategies) and molecular subgroup. Inherent limitations of the narrative design include the potential for selection bias in study inclusion and the absence of pooled statistical analyses.

## 3. First-Line Treatment

The incorporation of immunotherapy into first-line treatment has substantially transformed the management of advanced and recurrent endometrial cancer. Based on the results of recent pivotal phase III trials, the combination of immune checkpoint inhibitors with platinum-based chemotherapy has emerged as the preferred first-line standard of care in this setting, particularly following the results of RUBY, NRG-GY018, and AtTEnd [8,9,10]. These studies consistently demonstrated significant improvements in progression-free survival (PFS), with the greatest magnitude of benefit observed in patients with dMMR/MSI-H tumors, although clinically meaningful activity was also reported in pMMR disease.

Despite these advances, treatment benefit remains heterogeneous across molecular subgroups, and several important challenges persist, including optimal patient selection, treatment sequencing, duration of maintenance therapy, and management after progression on frontline chemoimmunotherapy. Consequently, current research efforts are increasingly focused on biomarker-driven strategies and rational therapeutic combinations aimed at overcoming resistance mechanisms and improving clinical outcomes, particularly in pMMR tumors, where intrinsic resistance to immunotherapy remains a major limitation.

### 3.1. Immunotherapy Plus Chemotherapy

Three pivotal randomized phase III trials (RUBY [8], NRG-GY018 [9], and AtTEnd [10]) have evaluated the incorporation of immune checkpoint inhibitors into carboplatin–paclitaxel chemotherapy in the first-line treatment of advanced or recurrent EC. Collectively, these studies established chemoimmunotherapy as a major therapeutic advance, consistently demonstrating improvements in progression-free survival (PFS) compared with chemotherapy alone, with the magnitude of benefit varying according to molecular subgroup (Table 3).

In the RUBY trial [8], the addition of dostarlimab to platinum-based chemotherapy significantly improved both PFS and overall survival (OS), with particularly pronounced and durable benefit observed in patients with dMMR/MSI-H tumors. Similarly, NRG-GY018 [9] demonstrated a significant PFS benefit with pembrolizumab plus chemotherapy in both dMMR and pMMR populations, although treatment benefit was substantially greater in the dMMR subgroup. The AtTEnd trial [10] further reinforced the role of chemoimmunotherapy by showing improved PFS with atezolizumab in combination with carboplatin and paclitaxel, again with the most favorable outcomes observed in dMMR disease, while OS data remain immature.

Importantly, these trials collectively support chemoimmunotherapy as the current preferred first-line standard of care for advanced and recurrent EC, particularly in dMMR/MSI-H tumors, where responses are deeper and more durable. In contrast, although patients with pMMR disease also derive clinically meaningful benefit, treatment efficacy remains more modest and heterogeneous, reflecting the lower intrinsic immunogenicity of this subgroup and the persistence of primary resistance mechanisms.

These observations highlight the need for improved predictive biomarkers beyond MMR status and support the ongoing development of rational combination strategies aimed at enhancing antitumor immune responses in pMMR tumors.

### 3.2. Immunotherapy Plus PARP Inhibitors

Combination strategies integrating immune checkpoint inhibitors with PARP inhibitors have emerged as a promising therapeutic approach, particularly in pMMR tumors, which generally derive limited benefit from immunotherapy alone. The biological rationale for this strategy is based on the ability of PARP inhibition to increase genomic instability, enhance neoantigen generation, activate interferon signaling pathways, and potentially promote a more immunogenic tumor microenvironment, thereby sensitizing tumors to immune checkpoint blockade.

In the phase III DUO-E trial [11], the addition of durvalumab to carboplatin and paclitaxel, followed by maintenance durvalumab with or without olaparib, significantly improved progression-free survival compared with chemotherapy alone. Notably, the incorporation of olaparib appeared to provide additional benefit, particularly in the pMMR subgroup, suggesting a potential strategy to partially overcome the intrinsic resistance to immunotherapy commonly observed in these tumors.

Similarly, RUBY Part 2 reported encouraging results with the combination of dostarlimab and niraparib, demonstrating improved progression-free survival in both the overall and pMMR populations [12]. However, although these findings are promising, longer follow-up and additional confirmatory analyses are needed to better define their impact on overall survival, long-term toxicity, and optimal patient selection.

Importantly, these combinations should currently be considered emerging therapeutic strategies rather than fully established standards of care. Several unresolved questions remain, including the identification of predictive biomarkers, optimal sequencing strategies, duration of maintenance therapy, and management of cumulative toxicity associated with prolonged combination treatment.

Nevertheless, the encouraging activity observed in pMMR disease supports continued investigation of PARP inhibitor-based combinations as a rational strategy to enhance antitumor immunity and expand the clinical benefit of immunotherapy in molecular subgroups with lower intrinsic immunogenicity.

### 3.3. Immunotherapy Plus Antiangiogenic Therapy

The combination of pembrolizumab and lenvatinib has demonstrated substantial clinical activity in previously treated advanced EC, with significant improvements in progression-free survival and overall survival compared with chemotherapy in the second-line setting [13,14,15]. Based on these results, the phase III ENGOT-en9/LEAP-001 trial [13] evaluated this combination as first-line treatment compared with standard carboplatin–paclitaxel chemotherapy.

However, lenvatinib plus pembrolizumab did not demonstrate a statistically significant improvement in progression-free survival or overall survival in the overall study population compared with platinum-based chemotherapy [13]. These findings suggest that, despite its established efficacy in previously treated disease, this regimen did not demonstrate superiority over current chemoimmunotherapy-based first-line strategies in unselected patients with advanced or recurrent EC.

Importantly, the negative results of LEAP-001 further reinforce platinum-based chemoimmunotherapy as the preferred first-line standard of care in advanced disease. Nevertheless, pembrolizumab plus lenvatinib continues to represent a key therapeutic option in later treatment lines, particularly in patients with pMMR tumors progressing after prior platinum-based therapy and without previous exposure to immunotherapy.

Additional analyses may help identify specific molecular or clinical subgroups that could derive greater benefit from antiangiogenic and immunotherapy combinations. Furthermore, treatment-related toxicity remains an important consideration with this regimen, given the high incidence of hypertension, fatigue, gastrointestinal adverse events, and dose modifications observed in clinical practice.

### 3.4. Immunotherapy in High-Risk Early-Stage Disease

The role of immunotherapy in high-risk early-stage endometrial cancer remains an area of active investigation. The phase III KEYNOTE-B21 trial [16] evaluated the addition of pembrolizumab to adjuvant chemotherapy, with or without radiotherapy, in patients with newly diagnosed high-risk disease. However, this study did not demonstrate a statistically significant improvement in progression-free survival compared with standard adjuvant treatment [16].

These findings suggest that, although immunotherapy has become an integral component of treatment in advanced and recurrent EC, its incorporation into the adjuvant setting remains uncertain and should currently be restricted to clinical trial settings. Ongoing studies are increasingly focused on biomarker-driven strategies aimed at identifying patient subgroups more likely to benefit from adjuvant immunotherapy, particularly according to molecular classification and tumor immune microenvironment characteristics.

Overall, the currently available phase III evidence supports platinum-based chemoimmunotherapy as the preferred first-line treatment strategy for advanced and recurrent EC. Although the magnitude of benefit is substantially greater in dMMR/MSI-H tumors, clinically meaningful activity has also been observed in pMMR disease, supporting the incorporation of immune checkpoint inhibitors into contemporary treatment algorithms.

## 4. Second-Line Treatment

The management of recurrent EC remains clinically challenging due to the increasing complexity of the therapeutic landscape and the growing incorporation of immunotherapy into earlier lines of treatment. In the second-line setting, therapeutic decision-making is primarily guided by prior exposure to immune checkpoint inhibitors, mismatch repair (MMR) status, platinum-free interval, disease burden, patient performance status, comorbidities, and previous treatment-related toxicities (Table 4). As a result, treatment selection has become increasingly individualized in routine clinical practice, particularly following the adoption of frontline chemoimmunotherapy strategies.

Despite recent therapeutic advances, several clinically relevant questions remain unresolved, particularly regarding optimal treatment sequencing after progression on prior immunotherapy. Currently, robust prospective evidence guiding post-chemoimmunotherapy management is limited, and treatment decisions frequently rely on molecular profile, duration of prior response, pattern of progression, toxicity considerations, and patient-related clinical factors.

Importantly, the therapeutic approach differs substantially according to MMR status and previous immunotherapy exposure. While PD-1 inhibitor monotherapy remains highly active in dMMR/MSI-H tumors without prior immunotherapy exposure, treatment options for patients with pMMR disease or acquired resistance after chemoimmunotherapy remain more limited and continue to represent a major unmet clinical need (Table 5).

### 4.1. Patients Without Prior Immunotherapy

In patients who have not previously received immunotherapy, immune checkpoint inhibitors represent a central therapeutic strategy, particularly in biomarker-selected populations. Anti-PD-1 agents such as pembrolizumab [17,18] and dostarlimab [19] have demonstrated clinically meaningful and often durable antitumor activity in advanced endometrial cancer, with objective response rates ranging from approximately 27% to 58% depending on molecular subgroup and study population.

In patients with dMMR/MSI-H tumors, PD-1-inhibitor monotherapy is currently considered a preferred second-line treatment following progression on platinum-based chemotherapy. Multiple clinical trials have demonstrated high response rates, durable disease control, and favorable tolerability in this subgroup, establishing pembrolizumab and dostarlimab as standard therapeutic options [20,21,22]. Given their relatively favorable efficacy-to-toxicity profile, anti-PD-1 monotherapies are particularly attractive in patients with low-volume disease, indolent progression, advanced age, frailty, or significant comorbidities.

In contrast, pMMR/MSS tumors generally exhibit lower intrinsic immunogenicity and limited responsiveness to single-agent immunotherapy, reflecting the presence of primary resistance mechanisms and a less inflamed tumor microenvironment. In this setting, the combination of lenvatinib plus pembrolizumab has become a major second-line treatment option. The phase III KEYNOTE-775 trial demonstrated significant improvements in progression-free survival, overall survival, and objective response rate compared with physician’s choice chemotherapy, with clinically meaningful benefit particularly in the pMMR population [15].

However, despite its efficacy, lenvatinib plus pembrolizumab is associated with substantial toxicity, including hypertension, fatigue, gastrointestinal adverse events, weight loss, and frequent dose modifications. Consequently, careful patient selection and close toxicity monitoring are essential in routine clinical practice. Factors such as performance status, comorbidity burden, frailty, prior treatment tolerance, and disease kinetics should be carefully considered when selecting candidates for combination therapy.

Therefore, lenvatinib plus pembrolizumab currently represents a preferred second-line strategy for patients with advanced or recurrent EC without prior immunotherapy exposure, particularly in the pMMR population and in patients considered suitable for combination treatment. In contrast, in dMMR/MSI-H tumors, PD-1-inhibitor monotherapy is generally preferred because of its high efficacy, durable responses, and more favorable toxicity profile. Nevertheless, the addition of lenvatinib may be considered in selected dMMR cases, particularly in patients with rapidly progressive, symptomatic, or high-volume disease, although this approach must be carefully balanced against the increased risk of treatment-related toxicity.

The platinum-free interval also remains an important factor in therapeutic decision-making. A retrospective analysis [23] demonstrated improved overall survival among patients retreated with platinum-based chemotherapy after a platinum-free interval of at least 6 months. Accordingly, platinum rechallenge may be considered in selected patients with late relapse [24,25], particularly in those who previously demonstrated platinum sensitivity and maintain adequate performance status. Emerging evidence also suggests that rechallenge with carboplatin and paclitaxel, with or without anti-PD-1/PD-L1 therapy, could represent a feasible strategy in selected cases [26], although prospective validation remains limited.

Beyond immunotherapy-based strategies, conventional systemic therapies continue to play a relevant role in selected clinical scenarios. Single-agent chemotherapy—including weekly paclitaxel, docetaxel, oxaliplatin, liposomal doxorubicin, topotecan, or ifosfamide—may be considered in patients with contraindications to immunotherapy, poor performance status, substantial frailty, or limited tolerance for combination regimens [27]. Hormonal therapy also remains a reasonable option in carefully selected patients, particularly in hormone receptor-positive tumors, low-grade histology, or indolent disease characterized by slow tumor progression [28].

### 4.2. Patients Previously Treated with Immunotherapy: Immunotherapy Rechallenge

With the increasing incorporation of immune checkpoint inhibitors into first-line treatment, disease progression following prior immunotherapy exposure is becoming progressively more common, representing a major unmet clinical challenge in advanced endometrial cancer.

At present, there is no robust prospective evidence supporting immunotherapy rechallenge outside the context of clinical trials. Available data remain limited and are largely derived from retrospective series and small prospective studies, which provide insufficient guidance regarding optimal retreatment strategies, sequencing approaches, or patient selection [29]. Consequently, no standardized therapeutic algorithm has yet been established for patients progressing after frontline chemoimmunotherapy.

Several clinical and biological factors may influence the likelihood of benefit from immunotherapy rechallenge, including duration of prior response, pattern and timing of progression, treatment-free interval, disease burden, and overall patient condition. Patients who previously achieved prolonged and durable disease control with immune checkpoint inhibition may represent a subgroup more likely to benefit from retreatment strategies; however, this hypothesis requires prospective validation.

Resistance to immune checkpoint blockade remains one of the principal limitations in this setting and is driven by complex and multifactorial mechanisms involving both tumor-intrinsic alterations and changes within the tumor microenvironment. Potential resistance mechanisms include impaired antigen presentation, alternative immune checkpoint activation, T-cell exhaustion, immunosuppressive cytokine signaling, and increased infiltration by regulatory T cells and myeloid-derived suppressor cells. Consequently, rational combination strategies integrating ICIs with targeted therapies, antiangiogenic agents, PARP inhibitors, or other immunomodulatory strategies are currently under active investigation in an attempt to restore antitumor immune activity and overcome acquired resistance.

Nevertheless, the optimal sequencing and clinical integration of these strategies remain poorly defined. In the absence of high-level evidence, enrolment in clinical trials should be strongly encouraged whenever feasible. Outside clinical trials, treatment decisions should be individualized and based on careful assessment of potential clinical benefit, cumulative toxicity, prior treatment tolerance, patient preferences, molecular profile, and disease kinetics [29].

## 5. Immune-Related Adverse Events

Immune checkpoint inhibitors (ICIs) are associated with a distinct spectrum of toxicities known as immune-related adverse events (irAEs), which result from nonspecific activation of the immune system. These events can occur at any time during treatment or even after its discontinuation, most commonly within the first 1–6 months of therapy, although delayed presentations have also been reported [30].

irAEs can affect virtually any organ system, most frequently involving the skin, gastrointestinal tract, endocrine glands, and liver [30]. The incidence and severity of these toxicities vary according to the class of ICI. In general, anti-CTLA-4 agents (such as ipilimumab) are associated with higher rates of both overall and severe adverse events compared with anti-PD-1 and anti-PD-L1 therapies. Reported rates of grade 3–4 toxicities are approximately 21.5% for anti-CTLA-4 agents, compared with 7.1% for anti-PD-1 and lower rates for anti-D-L1 inhibitors. Additionally, anti-PD-1 agents have been associated with a higher risk of colitis compared with anti-PD-L1 therapies (relative risk 1.80, 95% CI 1.22–2.67) [31].

Although fatal irAEs are uncommon, they may occur and require prompt recognition and appropriate management. Reported mortality rates are approximately 0.36% for anti-PD-1, 0.38% for anti-PD-L1, 1.08% for anti-CTLA-4, and up to 1.23% for combination regimens [32]. The spectrum of fatal toxicities varies according to the mechanism of action, with gastrointestinal events more commonly observed with anti-CTLA-4 agents, and pneumonitis, hepatitis, and neurologic toxicities more frequently associated with anti-PD-1/PD-L1 inhibitors. Cardiac toxicities, although rare, are particularly severe, with myocarditis associated with mortality rates approaching 40% [32].

Early recognition and appropriate management of irAEs are critical to minimize morbidity and mortality. Management strategies are based on severity grading and typically involve treatment interruption and immunosuppressive therapy. Mild toxicities may be managed with symptomatic treatment and close monitoring, whereas moderate to severe irAEs generally require temporary or permanent discontinuation of ICIs and initiation of systemic corticosteroids.

For grade 3–4 toxicities, current guidelines recommend high-dose corticosteroids (1–2 mg/kg/day), with gradual tapering once clinical improvement is achieved [33]. Importantly, the use of corticosteroids has not been shown to adversely affect oncologic outcomes in patients treated with ICIs [34]. In steroid-refractory cases, additional immunosuppressive agents such as infliximab or other targeted therapies may be required, depending on the organ involved and clinical context [34].

Given the potential severity and multisystem nature of irAEs, a multidisciplinary approach is essential for optimal management. Patient education, early symptom reporting, and close clinical monitoring are key components to ensure timely intervention and improve overall treatment safety.

## 6. Challenges and Future Directions

Despite the major therapeutic advances achieved with the incorporation of immunotherapy into the management of endometrial cancer, several important clinical and biological challenges remain unresolved. Although immune checkpoint inhibitors have substantially improved outcomes, particularly in dMMR/MSI-H tumors, a considerable proportion of patients (especially those with pMMR disease) continue to derive limited and heterogeneous benefit. These observations highlight the persistent challenge of primary and acquired resistance and underscore the need for more effective and biologically refined therapeutic strategies.

One of the principal unmet needs is the identification of predictive biomarkers beyond MMR status. Although MMR deficiency remains the most clinically relevant biomarker for response to immune checkpoint blockade, it does not fully capture the complexity of tumor immunogenicity or treatment resistance. Emerging biomarkers, including tumor mutational burden, *POLE* mutations, TP53 alterations, immune-related gene signatures, and characteristics of the tumor microenvironment, may contribute to improved patient stratification and therapeutic personalization. However, despite strong biological rationale, their clinical utility remains insufficiently validated for routine clinical practice and requires prospective confirmation [35,36].

Another major challenge involves optimization of treatment sequencing and duration within an increasingly complex therapeutic landscape. As chemoimmunotherapy becomes progressively integrated into frontline treatment [8,9,10], the management of patients progressing after prior immunotherapy exposure is becoming increasingly challenging. Currently, there is limited prospective evidence to guide sequencing strategies, retreatment approaches, maintenance duration, or the optimal integration of subsequent therapies following progression on frontline immune checkpoint blockade.

These challenges have driven the development of rational combination strategies integrating immunotherapy with chemotherapy, antiangiogenic agents, PARP inhibitors, and other targeted therapies to overcome resistance mechanisms. However, the optimal integration of these strategies into clinical practice remains uncertain and requires careful consideration of cumulative toxicity, biomarker-driven patient selection, treatment sequencing, and treatment-related adverse events.

Ongoing phase III trials are actively evaluating novel combination strategies aimed at expanding the benefit of immunotherapy to broader patient populations. In advanced disease, studies such as RUBY Part 2 and DUO-E are investigating the incorporation of PARP inhibitors into chemoimmunotherapy backbones, with encouraging preliminary results, particularly in pMMR tumors [12]. Nevertheless, although these findings are promising, these approaches should currently be considered emerging therapeutic strategies rather than fully established standards of care. Longer follow-up, confirmatory analyses, and biomarker-driven studies will be necessary to better define the magnitude of benefit and identify patients most likely to respond.

Although both anti-PD-1 and anti-PD-L1 agents have demonstrated clinical activity in endometrial cancer, indirect cross-trial comparisons should be interpreted with caution because of important differences in trial design, patient populations, molecular subgroup distribution, and treatment settings. Consequently, current evidence does not support definitive conclusions regarding superiority between these therapeutic classes. Nevertheless, anti-PD-1 agents currently have the strongest clinical evidence and the broadest FDA and EMA approvals in endometrial cancer, particularly in dMMR/MSI-H disease and chemoimmunotherapy combinations in advanced settings.

Beyond currently established approaches, emerging cellular therapies such as CAR-T and CAR-NK cells represent a promising area of translational research in gynecologic oncology. Although clinical evidence in endometrial cancer remains limited, preclinical studies suggest that these strategies may have future potential, particularly when combined with immune checkpoint blockade or other biologic therapies targeting the tumor microenvironment.

In the adjuvant setting, biomarker-driven strategies are increasingly being developed to individualize treatment in patients with high-risk early-stage disease. The RAINBO platform represents a paradigm shift toward molecularly tailored therapy, incorporating subtype-specific approaches such as maintenance olaparib in p53-abnormal tumors and immunotherapy combined with radiotherapy in dMMR disease [37]. These approaches aim to optimize treatment individualization while minimizing overtreatment (Table 6).

**Figure 2:**
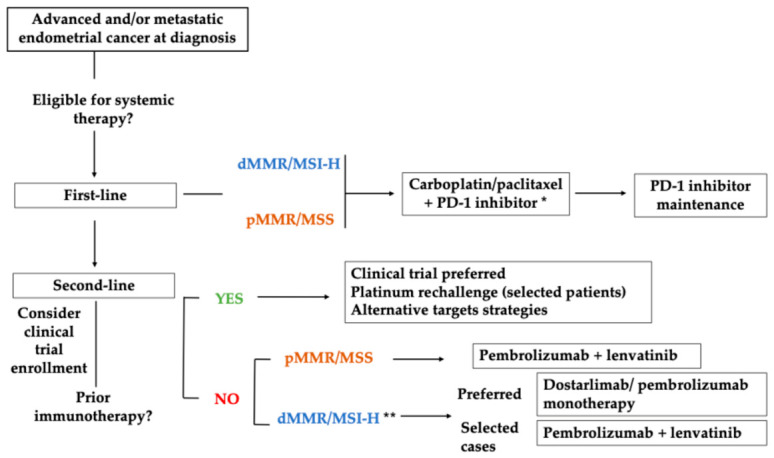
Clinical workflow for the management of patients with advanced or metastatic endometrial cancer at diagnosis eligible for systemic therapy. ∗ First-line regimen selection: Approved options include dostarlimab or pembrolizumab in combination with carboplatin and paclitaxel, in accordance with regulatory approvals (FDA/EMA) and clinical trial evidence (e.g., RUBY, NRG-GY018, and AtTEnd). Maintenance treatment duration varied according to trial protocol and was generally administered for up to 2 years or until disease progression. ∗∗ Management of ICI-naïve dMMR/MSI-H disease in the second line: Single-agent anti-PD-1 therapy (dostarlimab or pembrolizumab monotherapy) represents the preferred standard of care due to robust efficacy and favorable safety profiles. The combination of pembrolizumab plus lenvatinib remains reserved for selected clinical cases. For patients with pMMR/MSS disease who have not received prior immunotherapy, pembrolizumab plus lenvatinib is a valid second-line option. In the second-line setting, immunotherapy is generally continued until disease progression or unacceptable toxicity, although some trials, such as KEYNOTE-158, established a maximum treatment duration of up to 2 years. For patients progressing after first-line chemoimmunotherapy (Prior immunotherapy: YES), robust prospective data are currently limited; enrollment in clinical trials is highly preferred, with platinum rechallenge or alternative targeted strategies considered on an individualized patient-basis.

Finally, long-term outcomes, including durability of response, late toxicities, survivorship issues, and quality-of-life outcomes, remain insufficiently characterized, particularly in patients receiving prolonged immunotherapy exposure or combination treatment strategies. In this rapidly evolving field, the integration of molecular classification, biomarker development, and rational combination approaches will be critical to further advance precision immunotherapy and optimize outcomes for patients with EC. Figure 2 summarizes a clinically oriented treatment algorithm for advanced and recurrent endometrial cancer based on molecular classification, prior treatment exposure, and current evidence-based therapeutic approaches.

## 7. Conclusions

Immunotherapy has profoundly transformed the therapeutic landscape of endometrial cancer, particularly in advanced and recurrent disease. The incorporation of immune checkpoint inhibitors into first- and second-line treatment has resulted in clinically meaningful improvements in patient outcomes, especially in dMMR/MSI-H tumors, where durable and sustained responses are consistently observed.

The currently available phase III evidence supports platinum-based chemoimmunotherapy as the preferred first-line standard of care for advanced and recurrent endometrial cancer, particularly following the results of RUBY, NRG-GY018, and AtTEnd [8,9,10]. In contrast, although pembrolizumab plus lenvatinib has demonstrated substantial clinical benefit in previously treated disease [13,14,15], the negative results of LEAP-001 suggest that this combination does not currently replace chemoimmunotherapy-based strategies in the frontline setting for unselected patients.

Despite these advances, treatment benefit remains heterogeneous, particularly in pMMR disease, highlighting the limitations of current biomarker-driven strategies and the need for a deeper understanding of tumor biology, immune escape mechanisms, and the tumor microenvironment. In this context, rational combination strategies integrating immunotherapy with chemotherapy, antiangiogenic agents, PARP inhibitors, and other targeted therapies represent promising strategies to overcome resistance and expand clinical benefit to broader patient populations.

Although the results of DUO-E and RUBY Part 2 are encouraging, particularly in pMMR tumors, these approaches should currently be considered emerging therapeutic strategies rather than fully established standards of care [11]. Longer follow-up, confirmatory analyses, and biomarker-driven studies will be essential to better define the magnitude of benefit, identify patients most likely to respond, and clarify the optimal integration of PARP inhibitor-based combinations into routine clinical practice.

Several important challenges nevertheless remain unresolved, including optimal treatment sequencing after prior immunotherapy exposure, management of immune-related toxicities, identification of reliable predictive biomarkers beyond MMR status, and characterization of long-term outcomes and survivorship issues associated with prolonged immunotherapy exposure.

In this rapidly evolving field, future progress will depend on the integration of molecular classification, biomarker development, immune microenvironment characterization, and rational therapeutic sequencing strategies. These advances are expected to further refine precision immunotherapy and consolidate its role as a cornerstone of endometrial cancer management across disease stages.

## Figures and Tables

**Table 1:** Molecular classification and key features in endometrial cancer.

Molecular Subgroup	TCGA Subgroup	Key Features	Tumor Microenvironment	Prognostic Impact	Predictive Implications for Immunotherapy
*POLEmut*	*POLE* ultramutated	Ultra-mutated; high neoantigen load	Highly immunogenic	Excellent	Immunotherapy potential due to high neoantigen load
dMMR/MSI-H	Microsatellite instability–hypermutated	Hypermutated, MMR defects	Inflamed microenvironment with high TIL density, increased IFN-γ signaling, and PD-1/PD-L1 upregulation	Intermediate	Strong predictive biomarker to ICIs
NSMP	Copy-number low	Heterogeneous; no specific driver alterations	Variable immune infiltration and immune activation	Intermediate (heterogeneous; depends on additional risk factors)	Limited predictive biomarkers currently available; heterogeneous response
p53abn	Copy-number high (serous-like)	Chromosomal instability, frequent HER2 amplification (subset)	Immune “cold” phenotype with low T-cell infiltration	Poor	Candidates for targeted therapy (e.g., HER2 inhibition); limited but evolving role for immunotherapy

**Table 2:** Comparison between anti-PD-1 and anti-PD-L1 agents.

Characteristics	Anti-PD-1 Agents	Anti-PD-L1 Agents
Site of action	Bind to PD-1 expressed on activated T cells	Bind to PD-L1 on tumor cells and antigen-presenting cells
Mechanism of action	Block interaction between PD-1 and both PD-L1 and PD-L2	Block interaction between PD-L1 and PD-1
Effect on PD-L2 signaling	Inhibit PD-L2–PD-1 interaction	Do not interfere with PD-L2–PD-1 interaction
Representative agents	Nivolumab, pembrolizumab, dostarlimab, cemiplimab	Atezolizumab, durvalumab, avelumab
Immune-related adverse events	Similar toxicity profile (thyroiditis, colitis, pneumonitis, hepatitis)	Comparable immune-related adverse event profile
Current evidence in EC	Broadest clinical evidence and regulatory approvals in EC, particularly in dMMR/MSI-H disease and chemoimmunotherapy combinations	More limited clinical experience in EC, although activity has been demonstrated in combination strategies

**Table 3:** Phase III trials of ICIs in first-line advanced/metastatic endometrial cancer.

Trial	Experimental Arm	Primary Endpoints	n	Non-Endometrioid Histology (%)	Prior Adjuvant Chemotherapy Interval
NRG-GY018	Chemotherapy + pembrolizumab	PFS (dMMR, pMMR)	816	20%	>12 months
RUBY part 1	Chemotherapy + dostarlimab	PFS (dMMR, overall), OS	494	45%	>6 months
AtTEnd	Chemotherapy + atezolizumab	PFS (dMMR, overall) OS	549	34%	>6 months
LEAP-001	Pembrolizumab + lenvatinib	PFS (overall, pMMR) OS	842	33%	>6 months
DUO-E	Chemotherapy + durvalumab ± olaparib	PFS (durvalumab ± olaparib vs. control), OS	718	40%	>12 months

**Table 4:** Key second-line immunotherapy trials in advanced or recurrent endometrial cancer.

Study	Population	Treatment	ORR (%)	PFS (Months)	OS (Months)
KEYNOTE-158	dMMR/MSI-H	Pembrolizumab	48% (95% CI 37–60)	13.1 (95% CI 4.3–34.4)	Not reached
GARNET	dMMR	Dostarlimab	43.5% (95% CI 34–53)	Not mature	Not mature
GARNET	pMMR	Dostarlimab	14.1% (95% CI 0.1–20.6)	Not mature	Not mature
KEYNOTE-775	pMMR	Pembrolizumab + lenvatinib vs. chemotherapy	32.4% vs 15.1%	6.6 (95% CI 5.6–7.4)	17.4 (95% CI 14.2–19.9)
KEYNOTE-775	All-comers	Pembrolizumab + lenvatinib vs chemotherapy	33.8% vs 14.7%	7.2 (95% CI 5.7–7.6)	18.3 (95% CI 15.2–20.5)

**Table 5:** Current FDA and EMA approvals for immunotherapy-based treatments in advanced and recurrent EC.

Drug	Setting	Biomarker	FDA Approval	EMA Approval
Pembrolizumab + Carboplatin/Paclitaxel	Chemoimmunotherapy, 1L	All-comers	June 2024	October 2024
Dostarlimab + Carboplatin/Paclitaxel	Chemoimmunotherapy, 1L	dMMR/MSI-H	July 2023	October 2023
Dostarlimab + Carboplatin/Paclitaxel	Chemoimmunotherapy, 1L	All-comers	August 2024	January 2025
Pembrolizumab	Monotherapy, 2L	dMMR/MSI-H	May 2017	April 2022
Pembrolizumab + Lenvatinib	Combination, 2L	pMMR/MSS	September 2019/July 2021 *	November 2021
Dostarlimab	Monotherapy, 2L	dMMR/MSI-H	April 2021	April 2021

* Accelerated approval in 2019; full approval in 2021.

**Table 6:** Ongoing and recent phase III immunotherapy trials in EC.

Trial Name	ClinicalTrials.gov ID	Treatment Strategy	Clinical Setting	Primary Endpoint
NRG-GY020	NCT04214067	Pembrolizumab + radiation vs radiotherapy alone	Early-stage high-intermediate risk dMMR (I–II)	RFS
ENGOT-based trial (pMMR, TP53-mutated)	NCT07198074	Carboplatin-paclitaxel + pembrolizumab ± bevacizumab	Advanced or recurrent EC	PFS
KEYNOTE-B21/ENGOT-en11/GOG-3053	NCT04634877	Adjuvant pembrolizumab + chemotherapy ± radiotherapy	Newly diagnosed high-risk EC	PFS
RAINBO p53abn (Red Trial)	NCT05255653	Adjuvant chemotherapy + maintenance olaparib	High-risk p53abn EC	RFS
GCIG/DGOG/ENGOT-EN142 (Green Trial)	NCT05256225	Adjuvant radiotherapy + durvalumab	High-risk dMMR EC	RFS

## Data Availability

No new data were created or analyzed in this study. Data sharing is not applicable to this article.

## Abbreviations

The following abbreviations are used in this manuscript:

1L	First-line
2L	Second-line
CI	confidence interval
CTLA-4	cytotoxic T-lymphocyte-associated protein 4
dMMR	deficient mismatch repair
EC	endometrial cancer
EMA	European Medicines Agency
ENGOT	European Network of Gynaecological Oncological Trial groups
ESGO	European Society of Gynaecological Oncology
FDA	Food and Drug Administration
GCIG	Gynecologic Cancer InterGroup
GOG	Gynecologic Oncology Group
HER2 (ERBB2)	human epidermal growth factor receptor 2
ICIs	immune checkpoint inhibitors
IFN-γ	interferon gamma
irAEs	immune-related adverse events
MMR	mismatch repair
MSI-H	microsatellite instability–high
MSS	microsatellite stable
NSMP	no specific molecular profile
ORR	overall response rate
OS	overall survival
PARP	poly (ADP-ribose) polymerase
PD-1	programmed cell death protein 1
PD-L1	programmed death-ligand 1
PD-L2	programmed death-ligand 2
PFS	progression-free survival
pMMR	proficient mismatch repair
*POLEmut*	*POLE*-mutated
p53abn	p53 abnormal
RFS	recurrence-free survival
TCGA	The Cancer Genome Atlas
TILs	tumor-infiltrating lymphocytes
Tregs	regulatory T cells

## References

[1] Bray F., Laversanne M., Sung H., Ferlay J., Siegel R.L., Soerjomataram I., Jemal A. (2024). Global cancer statistics 2022: GLOBOCAN estimates of incidence and mortality worldwide for 36 cancers in 185 countries. CA Cancer J. Clin..

[2] Crosbie E.J., Kitson S.J., McAlpine J.N., Mukhopadhyay A., Powell M.E., Singh N. (2022). Endometrial cancer. Lancet.

[3] Levine D.A., The Cancer Genome Atlas Research Network (2013). Integrated genomic characterization of endometrial carcinoma. Nature.

[4] León-Castillo A., Gilvazquez E., Nout R., Smit V.T., McAlpine J.N., McConechy M., Commis S., van Weelden W.J., Evans D.G., Church D.N. (2020). Clinicopathological and molecular characterisation of “multiple-classifier” endometrial carcinomas. J. Pathol..

[5] Le D.T., Uram J.N., Wang H., Bartlett B.R., Kemberling H., Eyring A.D., Skora A.D., Luber B.S., Azad N.S., Laheru D. (2015). PD-1 Blockade in Tumors with Mismatch-Repair Deficiency. N. Engl. J. Med..

[6] Meric-Bernstam F., Makker V., Oaknin A., Oh D.Y., Banerjee S., González-Martín A., Jung K.H., Lugowska I., Manso L., Manzano A. (2024). Efficacy and Safety of Trastuzumab Deruxtecan in Patients with HER2-Expressing Solid Tumors: Primary Results from the DESTINY-PanTumor02 Phase II Trial. J. Clin. Oncol..

[7] Concin N., Matias-Guiu X., Cibula D., Colombo N., Creutzberg C.L., Ledermann J., Amant F., Asselain B., Battegay S., Berek J. (2025). ESGO–ESTRO–ESP guidelines for the management of patients with endometrial carcinoma: Update 2025. Lancet Oncol..

[8] Mirza M.R., Chase D.M., Slomovitz B.M., dePont Christensen R., Novák Z., Black D., Gilbert L., Sharma S., Tenney M.E., Vu K. (2023). Dostarlimab for Primary Advanced or Recurrent Endometrial Cancer. N. Engl. J. Med..

[9] Eskander R.N., Sill M.W., Beffa L., Moore R.G., Hope J.M., Musa F.B., Mannel R.S., Shahin M.S., Cantuaria G.H., Girda E. (2023). Pembrolizumab plus Chemotherapy in Advanced Endometrial Cancer. N. Engl. J. Med..

[10] Harano K., Fossati R., Pardo B., Galli F., Hudson E., Antill Y., Lee C., Rabaglio M., Heitz F., Kolovetsiou-Kreiner V. (2025). Phase III double-blind randomized placebo controlled trial of atezolizumab in combination with carboplatin and paclitaxel in women with advanced/recurrent endometrial carcinoma: The Asian cohort of the AtTEnd/ENGOT-EN7 trial. J. Gynecol. Oncol..

[11] Westin S.N., Moore K., Chon H.S., Lee J.Y., Thomes Pepin J., Sundborg M., Shai A., Deisback L., Kim J.W., Zamagni C. (2024). Durvalumab Plus Carboplatin/Paclitaxel Followed by Maintenance Durvalumab With or Without Olaparib as First-Line Treatment for Advanced Endometrial Cancer: The Phase III DUO-E Trial. J. Clin. Oncol..

[12] Powell M.A., Bjørge L., Willmott L., Novák Z., Black D., Gilbert L., Sharma S., Tenney M.E., Vu K., Secord A.A. (2024). Overall survival in patients with endometrial cancer treated with dostarlimab plus carboplatin-paclitaxel in the randomized ENGOT-EN6/GOG-3031/RUBY trial. Ann. Oncol..

[13] Marth C., Moore R.G., Bidziński M., Pignata S., Ayhan A., Rubio M.J., Powry J., Colombo N., Lorusso D., Herráez A.C. (2025). First-Line Lenvatinib Plus Pembrolizumab Versus Chemotherapy for Advanced Endometrial Cancer: A Randomized, Open-Label, Phase III Trial. J. Clin. Oncol..

[14] Eskander R.N., Sill M.W., Beffa L., Moore R.G., Hope J.M., Musa F.B., Mannel R.S., Shahin M.S., Cantuaria G.H., Girda E. (2025). Pembrolizumab plus chemotherapy in advanced or recurrent endometrial cancer: Overall survival and exploratory analyses of the NRG GY018 phase 3 randomized trial. Nat. Med..

[15] Makker V., Colombo N., Casado Herráez A., Monk B.J., Mackay H., Santin A.D., Miller D.S., Moore R.G., Baron-Hay S., Ray-Coquard I. (2023). Lenvatinib Plus Pembrolizumab in Previously Treated Advanced Endometrial Cancer: Updated Efficacy and Safety from the Randomized Phase III Study 309/KEYNOTE-775. J. Clin. Oncol..

[16] Van Gorp T., Cibula D., Lv W., Backes F., Ortaç F., Hasegawa K., Lindemann K., Savarese A., Laenen A., Kim Y.M. (2024). ENGOT-en11/GOG-3053/KEYNOTE-B21: A randomised, double-blind, phase III study of pembrolizumab or placebo plus adjuvant chemotherapy with or without radiotherapy in patients with newly diagnosed, high-risk endometrial cancer. Ann. Oncol..

[17] Marabelle A., Le D.T., Ascierto P.A., Di Giacomo A.M., De Jesus-Acosta A., Delord J.P., Geva R., Gottfried M., Penel N., Hansen A.R. (2020). Efficacy of Pembrolizumab in Patients with Noncolorectal High Microsatellite Instability/Mismatch Repair-Deficient Cancer: Results from the Phase II KEYNOTE-158 Study. J. Clin. Oncol..

[18] Ott P.A., Bang Y.J., Berton-Rigaud D., Elez E., Pishvaian M.J., Rugo H.S., Puzanov I., Mehnert J.M., Aung K.L., Lopez J. (2017). Safety and Antitumor Activity of Pembrolizumab in Advanced Programmed Death Ligand 1-Positive Endometrial Cancer: Results from the KEYNOTE-028 Study. J. Clin. Oncol..

[19] Oaknin A., Tinker A.V., Gilbert L., Samouëlian V., Mathews C., Brown J., Barretina-Ginesta M.P., Moreno V., Gravina A., Abdeddaim C. (2020). Clinical Activity and Safety of the Anti-Programmed Death 1 Monoclonal Antibody Dostarlimab for Patients with Recurrent or Advanced Mismatch Repair-Deficient Endometrial Cancer: A Nonrandomized Phase 1 Clinical Trial. JAMA Oncol..

[20] O’Malley D.M., Bariani G.M., Cassier P.A., Marabelle A., Hansen A.R., De Jesus Acosta A., Miller W.H., Riddle J.K., Topalian S.L., Schellens J.H. (2022). Pembrolizumab in Patients with Microsatellite Instability-High Advanced Endometrial Cancer: Results from the KEYNOTE-158 Study. J. Clin. Oncol..

[21] Makker V., Colombo N., Casado Herráez A., Santin A.D., Colomba E., Miller D.S., Smith F.A., Baron-Hay S., Rufián M.H., Lorusso D. (2022). Lenvatinib plus Pembrolizumab for Advanced Endometrial Cancer. N. Engl. J. Med..

[22] Oaknin A., Gilbert L., Tinker A.V., Brown J., Mathews C., Press J., Sabatier R., Kurtz J.E., Ferguson S.E., Boni V. (2022). Safety and antitumor activity of dostarlimab in patients with advanced or recurrent DNA mismatch repair deficient/microsatellite instability-high (dMMR/MSI-H) or proficient/stable (MMRp/MSS) endometrial cancer: Interim results from GARNET-a phase I, single-arm study. J. Immunother. Cancer.

[23] Moore K.N., Tian C., McMeekin D.S., Thigpen J.T., Randall M.E., Gallion H.H. (2010). Does the progression-free interval after primary chemotherapy predict survival after salvage chemotherapy in advanced and recurrent endometrial cancer?: A Gynecologic Oncology Group ancillary data analysis. Cancer.

[24] Miller D.S., Filiaci V.L., Mannel R.S., Cohn D.E., Matsumoto T., Tewari K.S., DiSilvestro P.A., Pearl M.L., Argenta P.A., Powell M.A. (2020). Carboplatin and Paclitaxel for Advanced Endometrial Cancer: Final Overall Survival and Adverse Event Analysis of a Phase III Trial (NRG Oncology/GOG0209). J. Clin. Oncol..

[25] Fleming G.F., Brunetto V.L., Cella D., Look K.Y., Reid G.C., Munkarah A.R., Fowler J.M., Burger R.A., Rubin S.C., Cullen M. (2004). Phase III trial of doxorubicin plus cisplatin with or without paclitaxel plus filgrastim in advanced endometrial carcinoma: A Gynecologic Oncology Group Study. J. Clin. Oncol..

[26] Bujnak C.A., File B., Tewari K.S. (2025). Clinical Use of Dostarlimab in Advanced Stage and Recurrent Endometrial Cancer: Patient Selection and Perspectives. Cancer Manag. Res..

[27] Eisinger C., Muluneh B. (2023). Combined use of pembrolizumab and lenvatinib: A review. J. Oncol. Pharm. Pract..

[28] Konstantinopoulos P.A., Lee E.K., Xiong N., Krasner C., Campos S., Kolin D.L., Liu J.F., Horowitz N., Wright A.A., Bouberhan S. (2023). A Phase II, Two-Stage Study of Letrozole and Abemaciclib in Estrogen Receptor-Positive Recurrent Endometrial Cancer. J. Clin. Oncol..

[29] Podder V., Coleman R.L., Eskander R.N., Flint M., Monk B.J., Slomovitz B.M. (2025). Checkpoint inhibitor rechallenge in advanced endometrial cancer: Revisiting the immune landscape beyond first-line therapy. Int. J. Gynecol. Cancer.

[30] El Osta B., Hu F., Sadek R., Chintalapally R., Tang S.C. (2017). Not all immune-checkpoint inhibitors are created equal: Meta-analysis and systematic review of immune-related adverse events in cancer trials. Crit. Rev. Oncol. Hematol..

[31] Miyashita H., Mikami T., Satoi S., Cruz C., Galsky M.D. (2020). Incidence and Risk of Colitis with Programmed Death 1 Versus Programmed Death Ligand 1 Inhibitors for the Treatment of Cancer. J. Immunother..

[32] Wang D.Y., Salem J.E., Cohen J.V., Chandra S., Menzer C., Ye F., Zhao S., Das S., Beckermann K.E., Reisman L. (2018). Fatal Toxic Effects Associated with Immune Checkpoint Inhibitors: A Systematic Review and Meta-analysis. JAMA Oncol..

[33] Petrelli F., Signorelli D., Ghidini M., Ghidini A., Pizzutilo E.G., Ruggieri L., Cabiddu M., Hanania A.N., Khakoo A.U., Brighenti M. (2020). Association of Steroids use with Survival in Patients Treated with Immune Checkpoint Inhibitors: A Systematic Review and Meta-Analysis. Cancers.

[34] Thompson J.A., Schneider B.J., Brahmer J., Andrews S., Armand P., Bhatia S., Budde L.E., Costa L., Davies M., Ingham M. (2019). Management of Immunotherapy-Related Toxicities, Version 1.2019. J. Natl. Compr. Cancer Netw..

[35] Lian M., Zhang C., Li T., Wang A. (2026). Immunotherapy in endometrial cancer: Mechanisms, clinical evidence, and future directions. Front. Immunol..

[36] Richardson M., Chase D.M. (2024). Latest advances in immuno-oncology for endometrial cancer: Single-agent and combination regimens. Curr. Opin. Obstet. Gynecol..

[37] Concin N. (2023). Refining adjuvant treatment in endometrial cancer based on molecular features: The RAINBO clinical trial program. Int. J. Gynecol. Cancer.

